# Time to consider animal data governance: perspectives from neuroscience

**DOI:** 10.3389/fninf.2023.1233121

**Published:** 2023-08-29

**Authors:** Damian Eke, George Ogoh, William Knight, Bernd Stahl

**Affiliations:** ^1^Centre for Computing and Social Responsibility, De Montfort University, Leicester, United Kingdom; ^2^School of Computer Science, University of Nottingham, Nottingham, United Kingdom

**Keywords:** animal research, animal data, neuroscience, data governance, ethics dumping, regulations

## Abstract

**Introduction:**

Scientific research relies mainly on multimodal, multidimensional big data generated from both animal and human organisms as well as technical data. However, unlike human data that is increasingly regulated at national, regional and international levels, regulatory frameworks that can govern the sharing and reuse of non-human animal data are yet to be established. Whereas the legal and ethical principles that shape animal data generation in many countries and regions differ, the generated data are shared beyond boundaries without any governance mechanism. This paper, through perspectives from neuroscience, shows conceptually and empirically that there is a need for animal data governance that is informed by ethical concerns. There is a plurality of ethical views on the use of animals in scientific research that data governance mechanisms need to consider.

**Methods:**

Semi-structured interviews were used for data collection. Overall, 13 interviews with 12 participants (10 males and 2 females) were conducted. The interviews were transcribed and stored in NviVo 12 where they were thematically analyzed.

**Results:**

The participants shared the view that it is time to consider animal data governance due to factors such as differences in regulations, differences in ethical principles, values and beliefs and data quality concerns. They also provided insights on possible approaches to governance.

**Discussion:**

We therefore conclude that a procedural approach to data governance is needed: an approach that does not prescribe a particular ethical position but allows for a quick understanding of ethical concerns and debate about how different positions differ to facilitate cross-cultural and international collaboration.

## 1. Introduction

In the last decade, the need to ensure reproducibility of research results and to justify public investment in research has led to increased sharing of research data and the imperative for open sharing. Open data platforms supported by research projects have increasingly become the center piece for facilitating open sharing of research data. In neuroscience, a number of these open platforms exist and share big, multitype and multifunctional data from diverse species of organisms for both research and innovation. As Poldrack and Gorgolewski (2014) pointed out, these platforms not only encourage data re-use and increase statistical power to stimulate translational knowledge but also expand the reach and impact of neuroscience research. A critical implication of this data re-use expansion is that data increasingly interacts with different jurisdictions with different regulatory requirements. While most of these datasets are from human participants, many are generated from animals.

Unlike human data that is nationally or regionally regulated (e.g., EU General Data Protection Regulation^1^), there are no established legal frameworks that govern the sharing and re-use of animal data nationally or internationally. One reason for this is that the sharing or use of animal data does not raise the traditional data use concerns associated with human data (Stahl et al., 2019) such as; privacy, fairness, human rights and security. The ethical and legal issues around animal data are usually raised during data creation. The scientific, ethical and legal validity of animal research data are mostly determined by the nature of the research procedure/experiment. The moral and legal questions of animal experiments do not always revolve around the implications of animal data usage but on the ethical and legal permissibility of its scientific generation. Crucially, the regulatory and ethical principles that shape animal data generation in many countries and regions are different while the generated data are shared beyond boundaries without any governance mechanism. This means that animal data generated in less restrictive places are openly shared in countries with very restrictive requirements mostly through open data platforms. Thus, this paper asks the question: is it time to consider animal data governance?

The paper shows conceptually and empirically that there is a need for animal data governance that is informed by ethical concerns. Animal data raises different ethical concerns from human data and thus needs to be treated differently. There is a plurality of ethical perspectives on the use of animals in research which any data governance regime will need to take into account. This article therefore arrives at the conclusion that a procedural approach to data governance is called for that does not prescribe a particular ethical position but allows for a quick understanding of ethical concerns and debate about how different positions differ to facilitate cross-cultural and international collaboration.

We organize this paper as follows. We explore the current international data governance ecosystem for responsible biomedical research and innovation; the continued use of animals for neuroscience research, especially non-human primates. We then provide a thematic analysis of interviews conducted with international neuroscientists who conduct animal experiments. On this basis we arrive at the conclusion that a procedural approach to international data governance is called for.

## 2. The continued use of animals in neuroscience research

Despite the increasing requirements to implement the 3Rs (replacement, refinement, and reduction) (Russell and Burch, 1960; Guhad, 2005), the use of animals in research continues in many parts of the world, especially in neuroscience research (perhaps more than in any other field of biomedical research) (Jones, 2021). Although public interest in the use of animals for research has significantly reduced the number of animal experiments in Europe and North America (Lankau et al., 2014; European Commission, 2019), animals continue to be central to scientific research in other parts of the world. The rationale for this is varied. Neuroscience research often involves invasive and non-invasive methods that cannot be conducted with humans because of associated risks and ethical concerns. Thus, neuroscientists turn to other animals such as; rodents, ferrets, dogs, pigs, zebra fish and monkeys whose usage in scientific research comes with considerably fewer ethical concerns. The argument for this, borders on the lack of safe and non-invasive approaches to studying the human brain (Preuss, 2010). Rodents are widely used because of their short gestation periods, their cost-effective production and have proved unquestionably effective (Neuhaus, 2018). Fruit flies (Zweier et al., 2009) and zebrafish (Haesemeyer and Schier, 2015) have also proved to be important research resources for neuroscience research.

The use of animals in invasive and intrusive neuroscience research is generally informed by the legal and ethical restrictions of such research with human brains. However, many neuroscientists suggest that there are anatomical and genetic limitations/differences that hamper the scope of their use (Garner, 2014; Windisch, 2014). These animal subjects or models present challenges Kaiser and Feng (2015) referred to as the lack of face validity and predictive validity. The delays in the development of new interventions for brain diseases are associated with the differences in pathophysiological mechanisms between rodents and humans (Ting and Feng, 2013). Differences in brain functions and cognitive behavior, difficulties in scalability of dosage regimens, differences in recovery times and differences in the ratio of white to gray matter in the brain are some of the issues that inform this lack of transnationality (Varki, 2000; Weatheall, 2015). For better predictive validity, some neuroscientists assert that non-human primates (NHPs) are particularly better subjects.

### 2.1. NHPs in neuroscience

Available draft genome sequences of primates have shown that there are important similarities between human and NHP genomes. NHPs have, indeed, been revealed as our closest relatives with regard to the DNA sequence of our genome (Li and Saunders, 2005). While the Chimpanzee genome is 98.77% similar to the human genome (Chimpanzee Sequencing and Analysis Consortium, 2005), the rhesus macaque has a 93.5% similarity (Disotell and Tosi, 2007). This phylogenetic proximity to humans and related similarities in anatomy, physiology and behavior form the basis for justification of the use of NHPs in neuroscience experiments (Tardif et al., 2013; Friedman et al., 2017). A further comparative study of primate genomics has also shown that the most significant evolutionary change between primates happened in the brain (Sikela, 2006). These similarities form the basis of justification and a remarkable curiosity to unlock the brain’s complex structure and functions through experiments with NHPs. Today, NHPs are mainly used in basic/fundamental neurobiological research to explore how brain circuits contribute to human brain activities such as; perception, attention, memory and emotion (Bystron et al., 2006). In Europe, some neuroscientists are engaged in this type of research even though the number of invasive/intrusive experiments ongoing is not known (Scientific Committee on Health, Environmental and Emerging Risks [SCHEER], 2017). They are also used in translational and applied neuroscience research (Capitanio and Emborg, 2008) aimed at understanding the causes and development of potential treatments of brain disorders. Even though the scientific and translational validity of NHP experiments have been questioned (Knight, 2007; Bailey and Taylor, 2016), NHP research in neuroscience continues to develop new tools and approaches.

There is also a steady rise in neurological alterations of NHPs to model human brain diseases/functions and to study the genetic mechanisms that inform human specific neurological changes (Shi et al., 2019). This can be in the form of neural grafting (Bjugstad and Sladek, 2006), transgenesis (Chan, 2014) or other forms of human-NHP chimerism. These experiments significantly alter the neurobiological appearance, behavior or genetic makeup of the NHP causing phenotypic changes. Transgenesis refers to the artificial transfer of a foreign gene into the genome of another organism in order to introduce or delete characteristics of the phenotype (Mepham et al., 1998). In neuroscience, this can involve the use of HLS (human lineage specific) genes to create transgenic NHPs to demonstrate changes in brain structure (like brain size), function (such as high cognition) or to model diseases (like autism, Huntington diseases etc.). These genetic alterations of NHPs are developed with customized mutations and have shown to be able to model human brain disorders like Parkinson’s (Yun et al., 2015), Schizophrenia (Qiu and Li, 2017), Alzheimer’s (Yeo et al., 2015), autism (Cyranoski, 2016; Zhao et al., 2018) and Huntington’s disease (Tomioka et al., 2017). Another invasive NHP experiment in neuroscience involves the transplantation of human-derived neural cells into an NHP to model “human-like behavior”- neural grafting. These and similar neuroscience research experiments with NHPs present unique concerns. The controversy is not confined to the scientific community but extends to the wider public. But the fact that significant neurological similarities between humans and NHPs raise ethical concerns that needs attention and some agreement by many stakeholders (Conlee and Rowan, 2012; Carvalho et al., 2018). In essence, the unique usefulness of NHPs neuroscience research also shapes the unique ethical and legal questions they raise. Overall, this increases the imperative for animal data governance in neuroscience.

## 3. Current international data governance ecosystem

Data governance is defined as “the principles, procedures, frameworks, and policies that ensure acceptable and responsible processing of data at each stage of the data life cycle, from collection, storage, processing, curation, sharing, and use to deletion” (Eke D. O. et al., 2022). The emphasis on the data life cycle demonstrates that data governance is not only required for a specific stage of the life-cycle. It is a robust framework that starts before data collection and continues to the deletion stage. Fothergill et al. (2019) described it as the overall management of the availability, usability, integrity, quality, and security of data in order to ensure that the potential of the data is maximized while regulatory and ethical compliance is achieved within a specific organizational context. This definition introduces ethical compliance as an important aspect of data governance. Data governance is therefore more than legal compliance (Eke D. et al., 2022). It includes adherence to available ethical principles.

Furthermore, whereas the interpretations of data governance in organizations, disciplines and projects are different (Stahl et al., 2018), its goals and objectives are rooted in available laws and ethics. The question of whose laws and ethical values is determined by the context. Available regulations and ethical values are still jurisdictionally constrained while data continues to cross borders and socio-cultural contexts. For instance, data protection laws are established for specific jurisdictions (e.g., EU GDPR, USA HIPAA^2^, Canada’s PIPEDA^3^ etc.). Ethical values and principles that shape data governance also emerge from specific socio-cultural backgrounds. This means that the meaning or interpretations of data ethics principles such as trust, autonomy, privacy and consent are different in different cultures and societies. These inform relative interpretations of data governance in different cultures.

It is also important to note that established data related regulations and ethical narratives focus mainly on human data. The literature and practice of data ethics and data protection exists to address issues that affect humans in the data processing pipelines. To the best of our knowledge, there is no existing regulation established to address ethical, legal and societal issues related to animal data. This is true for both research and non-research settings. In their systematic literature review of ethical principles that shape data governance discourse in neuroscience, (Ochang et al., 2022) identified a number of ethical principles that often shape data governance discourse in brain research. None of the principles identified touched on animal data concerns. That means that data governance mechanisms often exclude animal data concerns as it relates to ethics and the law. Aspects of technical elements of animal data governance are, however, often included in discussions on findable, accessible, interoperable, reusable (FAIR) data principles. These include aspects of data standardization, integration and interoperability. This falls short of addressing ethical and legal concerns related to the different stages of animal data lifecycle while animal experiments continue to be critical parts of biomedical research.

## 4. Emerging interests in animal data governance in neuroscience

For a number of reasons, including socio-cultural, ethical and legal differences that inform what is considered permissible use of animals in research, there is a growing interest in the governance of animal data (Eke D. O. et al., 2022). Perceptions on ethical concerns about the use of animals, particularly NHPs, in biomedical research are fundamentally shaped by diverse socio-cultural norms, ethical principles and regulatory requirements. This is evident in the fact that while the use of NHPs in research has decreased significantly in Europe and America due to increased social animal welfare activism, NHPs are still the central focus of big neuroscience projects in Asia (Okano et al., 2016; Poo et al., 2016).

Advancements in genome-editing technologies such as the CRISPR/Cas system and artificial intelligence amplifies the huge possibilities in generating genetically modified NHPs. Although this emerging field (genetically modified NHP) has the “potential to transform the study of higher brain function and dramatically facilitate the development of effective treatment for human brain disorders” (Feng et al., 2020), it raises significant ethical concerns. Responding to the huge potential of genetically modified NHPs, Rommelfanger et al. (2018) raise the concern that researchers may be able to introduce cognitive capabilities that can contribute to blurring the boundaries of personhood and ultimately alter traditional perceptions of animal ethics. In 2019, a group of researchers from the Kunming Institute of Zoology in China claimed to have created transgenic monkeys with improved cognitive capacity (Shi et al., 2019). These modified monkeys were created with human MCPH1 genes and were not modeling any human diseases. They were simply modified to be phenotypically humanlike. During a series of cognitive tests, the researchers reported that these animals displayed better short-term memory than their counterparts in the wild. Basically, their brain development mirrored human brain development in many respects. The underlying logic behind this research, which is to manipulate monkeys to model humanlike capacities, presents a slippery slope concern. The question is where would the line be drawn in the path to generate human-like animals? To say the least, such research will not be permissible in many socio-cultural, ethical and legal contexts where the moral status of NHPs are hotly debated.

Given these differences in attitudes, values, principles, beliefs, regulations on the use of animals in research, Rommelfanger et al. (2018) further noted that, “sharing of brain data between countries that hold different ethical stances on what is considered appropriate animal experimentation raises additional questions.” One of such questions is; “Should a country accept or use data collected elsewhere in a fashion that is not considered locally ethical?” (Ibid). This is a critical question at the heart of animal data governance consideration. It presents an ethical dilemma many scientists currently face in the neuroscience data sharing ecosystem (Eke D. O. et al., 2022). One project that has stated this as a concern is the PRIMatE Data Exchange (PRIME-DE) Consortium that has highlighted the lack of international standards and regulations as barriers to fostering international NHP data sharing and collaborations (Milham et al., 2020). This increased interest in neuroscience deserves more exploration and hence this paper.

## 5. Methodology

The issue of responsible animal data governance requires multi-stakeholder perspectives and insights (Rommelfanger et al., 2018; Eke D. et al., 2022). It calls for the appreciation of diverse cultural values and beliefs while respecting established ethical frameworks. Thus, a semi-structured interview was selected as the methodological choice. The underlying research philosophy, therefore, is to use social actors to provide in-depth and rich perspectives on the reality of animal data governance. The aim was to provide detailed and reasoned insights rather than objectively generalisable positions.

The target population included researchers around the globe who have conducted or are conducting animal experiments to answer diverse neuroscience questions. Participants were drawn from active research projects under the International Brain Initiative (IBI). The IBI is the umbrella body for all the large-scale brain research initiatives including the EU Human Brain Project, the US Brain Initiative, Japan Brain/MINDS, Australian Brain Alliance, Korean Brain Initiative, Canadian Brain Research Strategy, and China Brain Project. We also drew participants from Africa and Latin America. Initial list of 37 researchers was compiled and interview invitations extended to all of them. A total of 15 people accepted the invitation and 3 later withdrew citing busy schedules. A structured interview guide that aligns well with the principles of qualitative research design (Ragin and Amoroso, 2011) was developed and tested on two colleagues. Following feedback from these initial tests, the interview protocol was improved before the start of the interviews.

Ethics approval was obtained for this research from De Montfort University ethics review committee. Information sheet and an informed consent form were then emailed to participants. The information sheet contained comprehensive data on the research including but not limited to the research objectives, expectations from the participants and responsibilities of researchers, potential risks and benefits, the voluntariness of participation and how research data will be used. All participants returned the consent form before the interviews and further verbal consent was sought at the start of the interview to record the session. The interviews were conducted either in person or virtually via Skype or zoom and took approximately 40 min each to complete. These interviews occurred between January 2020 and November 2021. Overall, 13 interviews with 12 participants (10 males and 2 females) were conducted. One participant was interviewed twice because the first interview was cut short due to technical difficulties. A total of 13 interviews were considered sufficient to achieve saturation because according to Guest et al. (2006), theoretical saturation can be achieved even in 12 interviews and basic elements for metathemes can emerge as early as six interviews.

The interviews were transcribed and stored in NviVo 12 where they were inductively coded for themes by the first author (DE). The inductive coding process involved reading through the transcript and identifying common patterns and themes. Thematically, the coding tree included high level nodes that show participants’ perspectives on why it is time to consider animal data governance or otherwise (such as cultural differences, legal differences etc.). Specific themes that align with high level themes are coded as sub-nodes. For example, *ethics dumping* was identified as a sub-node under the high-level node of *ethical differences*.

## 6. Key findings

One of the key results that emerged from the interviews was that all the participants agreed that due to the increasing transfer of animal data across countries, it is time to consider animal data governance for a number of reasons. The reasons provided by the participants are diverse and include: differences in regulations, socio-cultural and ethical values and beliefs, as well as scientific quality (see Table 1).

**TABLE 1:** Summary of key findings.

Reasons why it is time to consider animal data governance	
Differences in regulations	● Animal welfare is regulated differently in different countries and regions. ● While NHPs remain the most suitable animal for research in Neuroscience, their use in research are legally restricted in many countries. ● There are no identifiable existing laws in these countries (with restrictive laws) against the use of animal data. *However, in countries where animal experiments are legally not allowed, it should not be legal to use data that emerge from such experiments conducted in other countries.*
Differences in socio-cultural values, beliefs, attitudes, and ethics	● Socio-cultural values, belief systems, attitudes and ethics shape regulations on the use of animals in research. ● Whereas some cultures allow and even encourage the use of animals in research, other cultures actively work against the use of animals in research. ● Strict animal welfare regulations and social activism against the use of animals in research are pushing researchers to outsource ethically questionable experiments to countries where there are little or no laws–ethics dumping. *Animal data governance will help to prevent ethics dumping and ensure that socio-cultural values and ethics are adhered to.*
Data quality concerns	● Good science requires good data from reliable experiments. ● The quality of animal data depends on good animal care (overly stressed animals are bad subjects). *Animal data governance will help to ensure good animal care.*
**Approaches to animal data governance**	
● Requires a multi-stakeholder dialog for the co-creation of actionable frameworks (involving people from different cultures and animal). ● Comparative study of existing regulations and the socio-cultural values and beliefs underlying them. ● Can build on established minimum standards used for journal publications. ● Requires regulations similar to human data regulations. ● International research infrastructures where researchers meet data from different regions of the world will play in vital role in achieving effective animal data governance for research and innovation.	

### 6.1. Differences in regulations

A number of the participants pointed out legal differences as one of the major reasons why it is time to consider the governance of animal data. For instance, one participant stated that: “*although there is no regulation related to the use of animal data that I know of, there should be one since there are differences in regulations for the generation of animal data*”- (P2).

Another participant also confirmed that: “*Regulations inside laboratories around the world are not the same, why should the data we generate be treated the same way?*” (P1).

Furthermore, another participant presented these regulatory differences with an example of how it prevents collaborations: “*I have colleagues in the UK and we discussed collaborative projects but it turned out to be impossible because the Ethical Research Council’s standards do not match Japanese standards. On another occasion, how to use data obtained from Japan, because it was monkey data, was the major problem*” (P5).

To reiterate this angle of NHPs, one participant stated that: “*It is not just that Africa has the animals or the research is cheaper here but it is because that they are allowed to do in some African countries, especially with NHPs, they are not legally allowed to do in their own countries*” (P11).

The mention of non-human primate (NHP) here is critical because as pointed out above, differences in animal welfare are more amplified when it involves NHPs. These legal differences lead to an ethical question of how to share and use data generated from experiments legally not permissible in certain regions of the world.

Some participants raised further issues and concerns related to power imbalance that may emerge due to non-harmonized regulations. One participant observed that: “*If one country has a very loose standard, this country is capable of doing many things that could not be done in some other places. This group will dominate its power in the science of new data maybe. I think this is happening now. If there cannot be harmonized welfare regulations due to many factors, then we should have some open-minded policy discussions on responsible sharing of the datasets*” (P1).

This observation shows that differences in regulation are giving researchers in certain countries (with less strict provisions) scientific advantage over others. In responding to this, another participant stated that: “*We ran into that with stem cells, in the United States. And the argument was, ‘well, if you don’t fund it, they’re going to fund it over there, and we’re going to fall behind.’ but you cannot build your industries on the back of something that is ethically wrong*” (P3).

This was an important point since regulations are fundamentally shaped by societal values which are different in different regions. These findings align with evidence that has been demonstrated in literature. Vasbinder and Locke (2016) provided an overview of regulatory frameworks across the globe that demonstrated that whereas there are common standards across different jurisdictions, there are clear differences in how different countries regulate the use of animals in research. Most importantly, they identified that animal research is “performed in African and Middle Eastern countries, but many of these countries have not yet enacted legislation nor established regulatory oversight, policies or guidelines” (Vasbinder and Locke, 2016 p. 263). Mitchell et al. (2021) have also provided detailed insights on the differences in regulatory provisions guiding the use of primates in neuroscience across countries which is the focus of this research. Some of the differences they pointed out include but are not limited to; how NHPs should be generated for use in research (e.g., the use of wild NHPs for research purposes are banned in the EU, in China prior to COVID-19 pandemic this was allowed). Another difference they pointed out is the sizing and flooring of the home enclosures and caging. The EU provides that NHPs should be housed in larger sized enclosures and cages while in China and other Asian countries the cages are smaller (Mitchell et al., 2021). These differences have implications such as forming barriers to effective international collaborations and global data sharing (Rommelfanger et al., 2018; Milham et al., 2020).

### 6.2. Differences in ethical principles, values and beliefs

It has been established in literature that the diverse socio-cultural values, beliefs, attitudes and ethics found in many regions of the world greatly influence the available diverse animal welfare regulations (Masiga and Munyua, 2005; Szucs et al., 2012; Garcia and McGlone, 2022). The participants highlighted the conflicting cultural and religious beliefs that make animal data governance an imperative. As one participant put it:

“*I think policy of the animal ethics and welfare differ between countries…there are conflicting concepts and beliefs… and there should be respect for people’s cultures*” (P6).

Another observation was;

“*Our cultures are different. In our culture we believe that using animals for a scientific purpose is exactly the same as killing animals to eat to maintain our lives because the scientific research leads to the development of human medicine. That is culturally accepted. However, it is different in Europe*” (P5).

These opinions suggest that the acceptability of generation of animal research data is different in different societal contexts. There are differences in the understanding and conceptualization of ethical concerns associated with animal experiment. One of these concerns involves the idea of inflicting *pain* on animals. One participant stated that when scientists are inducing psychiatric disorders, they are creating suffering for the animals which is something that should be deeply examined. Reacting to pain associated with inducing psychiatric disorders in animals, a participant observed that; “*there are ethical concerns, and I think there should be a very deep and profound discussion about it*” (P7).

However, there was an opinion to always focus on the balance between costs and benefits of research experiments that cause pain. For this participant, “*cost and benefit balance is the most critical issue… such a kind of disease model can cause some painful situations in these animals and even this can also be an experiment for the chronic pain you know*” (P4).

Furthermore, the idea of pain and how it affects animals is amplified when NHPs are involved.

*The general belief that monkeys are better animals for experiments because they are closer to humans in cognitive abilities suggests that they will feel more pain than rats and others. This is what we have come to know…I don’t believe researchers in so many other places respect this. We know too much about primates, we know too much about their sociality, we know too much of this for us to use them as our own personal lab rats* (P8).

This is because NHPs are *sophisticated, capable entities* (P1). Therefore, we should be thinking about *minimizing suffering* (P1) rather than inflicting pain on them.

There are also heightened ethical concerns when transgenic NHPs are involved. According to a participant; *Technology has advanced and many tools are now available. You don’t want to create monster-killing animals that can be used for military purposes and things like that* (P9).

Similarly, one participant observed that advancement in technologies are improving research but may be used in unethical ways. *We do have the tools in our hands to start playing dangerous games. That is definitely the case, not only in non-human primates; even in humans. There are possibilities of creating subjects that we don’t want on earth. These experiments may be culturally allowed in some places and rejected in others. Governance of how the datasets that emerge from such dangerous experiments can deter such research* (P3).

For another participant responding to whether it is ethical to use data from transgenic NHPs, conditions under which such data is generated should be carefully examined because; *there are real concerns, especially as you get into transgenic models, to really consider whether or not you want to be seen endorsing a particular approach or not. That depends on where the researcher is working from* (P4).

To support this line of thought, another participant stated that: “*…this is a serious ethical concern. The use of the data from research that is not allowed in this country* (the participant’s country) *can cause reputational damage. I have not thought about it this way but it can*” (P10).

Another argument presented by the participants was that the differences in ethical principles, values and regulations means that some researchers will move to other countries with less restrictive values and regulations or in some cases outsource the research. These are insights provided by the participants:

*We already know that happens. We knew it happened with stem cells, we knew it happened with anything else, “…can’t do it here, I will go and do it in that country, where there are no regulations.” Some researchers can do the research in other countries and then bring back the data to be used here. This is data laundering and it is fraud, right? You are just outsourcing it to somebody else and taking advantage of the data* (P3).

*To be fairly honest, I think at some scale that’s already happening. There are people from Europe doing some kind of research in China for Example, which they would have a very difficult time to get easily approved in their own country. The same is true for the United States. So, in theory, yes, this opens the doors for that kind of stuff, but that’s not where we want to go* (P1).

Another participant observed that animal data governance can

*Prevent unnecessary animal research happening in Africa by researchers from Europe and America. So many of these do not follow the same ethical principles they are made to follow in their own countries* (P11).

These perspectives hint at an ethical concern often referred to as ethics dumping which will be explored further in sections below. It also means that while researchers in different regions of the world are required to comply with their regulations, some of these regulations may fall short of acceptable ethical standards in other regions.

### 6.3. Data quality concerns

Another reason the participants believe that it is time for animal data governance is to ensure that the quality of animal data shared is good for scientific purposes. The summary of the opinions here is that data governance can improve the quality of data because good scientific research relies upon high quality laboratory animal care (Friese, 2013, 2019). A harmonized animal data governance can help to improve animal welfare in a way that fewer confounding variables are introduced into research. Some of these opinions are as follows:

*But we also know that overly-stressed animals are bad subjects, you don’t get good data from them, you see mixed effects. So, I think you could make both the ethical and the scientific case that these animals need to be treated well. Governance can help harmonize best practices for animal welfare* (P4).

*It will be a con-founding factor. If there are high stress levels on the animals, it will simply provide you with completely wrong measurements of whatever you are doing. Yes, that is what I think. Having some sort of universal principle for the derived data will improve the quality of data shared* (P12).

*The quality of data is critical to this discussion. I think in science you want a certain degree of consistency and quality for effective research outcomes. Open data portals need to put mechanisms to ensure that the data they make available come from labs that comply with high standards of animal welfare* (P9).

These views suggest that animal data governance when implemented, particularly by open data repositories/archives, can help to improve the quality of animal data for research. However, without adequate governance mechanisms, low quality animal data may be allowed to permeate within research ecosystems.

### 6.4. Approaches to governance

Beyond providing insights into the reasons why it is time to consider animal data governance, the participants also gave their perspectives on how this can be done. One view that was shared by all the participants is that a harmonized governance framework for animal data requires inclusive discussions involving all relevant stakeholders. In order to appreciate and respect differences in regulations and social-cultural values, animal data governance is a multi-stakeholder endeavor. For instance, one view was that:

*Discussions on governance approaches should be thoughtful and careful and involve researchers that are performing these experiments. It should also involve people from different cultures to understand what is an appropriate framework* (P12).

This is a very important view that can ensure that one region does not dictate for other regions as regards best practices. As a participant mentioned; *responsible data governance is an interesting concept but a complex one. Whose understanding of responsibility? Whose values are going to shape this? These are things that need further discussions and understanding* (P5).

Another participant also observed that exclusion or disregard of some cultural values and beliefs will make any developed mechanism unacceptable for many scientists. However, this does not mean that people working in regions with strict regulations should accept anything and everything.

There was also the feeling that already established standards used for publishing in journals can be a starting point–something to build on.

*Minimum standards of acceptance and rejection for papers in neuroscience journals need to be studied and improved upon. For instance, the implementation of the 3Rs. Maybe a comparative literature about the different ethical standards used in different countries* (P2).

Another researcher further suggested that *auditing all data producing sites* can be a pragmatic way of understanding the *status quo*.

One critical view that was shared by many of the participants is that research infrastructures or open data archives, repositories or portals need to play important roles in providing efficient governance mechanisms for animal data. The argument was that animal data should be *governed through international research infrastructures where researchers and research data from different cultures meet* (P10).

Some participants made a case for the establishment of regulations for the use of animal data similar to data protection laws. These can be new regulations or amendments to existing laws to cover the use or application of animal data, particularly NHP data. An EU participant puts it thus:

*The rationale is, of course, that we wouldn’t include data that has been acquired in a way that doesn’t conform to the rules within the EU. That is important. Otherwise, it serves as an incentive, almost, for people to do something elsewhere and then hopefully get the data in* (P7).

## 7. Discussion and conclusion

The findings from our empirical work are broadly consistent with our insights from the existing literature. The international neuroscientists we talked to agreed that animal neuroscience research raises ethical concerns and that these concerns have consequences for the way the resulting data can and should be used. The very brief answer to the question in the title to this article whether it is time to consider animal data in data governance is thus a “yes.”

Our empirical work highlights that this is not a purely theoretical problem but that questions of animal data governance do arise in practical neuroscience work. The global discussion of data governance in health-related research ostensibly has the purpose of facilitating collaboration and exchange of data with a view to support the creation of new knowledge and the resulting consequences. This logic can be extended to animal data which calls for animal data governance.

There remain, however, fundamental differences between human data and animal data. The very use of human data can raise ethical concerns, for example where patient record privacy is concerned. Animal data does not raise such intrinsic concerns. For animal data the core of most issues is the generation or collection of data. Data use is nevertheless important because a lack of attention to the use of data may facilitate the use of data which is deemed not to be ethically permissible in a particular jurisdiction or cultural context, thereby sidestepping agreed-upon ethical principles.

A key question is therefore which type of animal research is deemed to be permissible and on what grounds can such value judgments be made. Our interviews showed that neuroscientists are aware of differences with regards to these questions. There seems to be a continuum of ethical severity which starts with cell cultures, moves up via invertebrates, vertebrates, rodents, NHPs and may find its current summit in research on transgenic NHPs. The problem is that the evaluation of these different types of research differ between cultures and jurisdictions and there is no universally agreed position on these questions.

This lack of agreement points to the lively exchange of ideas between cultures which is probably a good thing. Ethics is a topic that often finds its expressions in dilemmas and disagreements, so the plurality of views is not surprising. It seems plausible that an ethical free-for-all is not desirable, neither in animal research, nor in research more generally. At the same time, a uniform ethical position would suppress legitimate positions and thus be likely unethical in itself. Ethical plurality is thus welcome and can stimulate academic debate, for example in the field of neuroethics, where questions are continually triggered by new technologies and methods. While we thus welcome ethical plurality on animal research, it raises practical questions with regards to what data can and should be used for which purposes.

This leaves us with the question of how ethical pluralism can be accommodated in data governance. One plausible response to this question could come from procedural approaches to ethics. What this means is that we should not expect to find agreement on the substance of complex ethical questions, but it may be possible to define procedures that support fruitful engagement on these questions. One can argue that most modern western approaches to philosophical ethics follow such a procedural approach. Elsewhere we have suggested that Discourse Ethics may provide a suitable avenue to pursue (Stahl et al., 2019). We believe, without having the space to make this argument in detail, that such a procedural approach could be applied to the problem of animal data governance.

In practice this would mean that data governance should be designed in a way to facilitate constructive debate about ethical issues and be suitable for supporting ethical consensus, where it exists. This implies that data governance should be used to highlight ethically contentious aspects of animal data. This means that the meta-data of neuroscientific animal research data should clearly show those aspects that we know to be ethically contentious. This would include the species, research question, type of intervention, whether transgenic animals are involved etc.

The result of such an approach to animal data would be that it would be easy to understand ethical agreements and disagreements. If, for example, a culture has a consensus view that *in vivo* experiments with NHPs or the use of transgenic animals is not ethically acceptable, then filtering out such data would be easy. Probably more importantly, a strong metadata schema would allow highlighting which aspects of the research and the resulting data may be contentious and thus foster communication around the reasons for disagreements and possible ways to shape research and data in ways that are more broadly deemed to be acceptable.

This proposal is of course not overwhelmingly novel. Data governance structures already routinely capture some of these items. As our interviews have shown, researchers see data quality as an (ethical) issue and good metadata is recognized as a means to increase transparency of data and to promote the FAIR principles. The novelty of our proposal is that ethics-related aspects of the metadata could be explicitly defined and collected. While many of these will already form part of data governance, a next step would be to more clearly define them in ways that support researchers who generate the scientific data in the first place and ensure that they are aware of relevant metadata requirements.

This proposal is of course no panacea. There are limitations of our research such as the limited number of respondents and a lack of statistical representativeness of our approach. While we believe that our methodological choices have ensured that we received relevant input, we cannot claim to have represented all possible angles and identified all ethical issues. This article should thus be read as an exploratory study that has confirmed that ethically informed animal data governance is called for. The real work of shaping such a data governance approach will have to follow as a large-scale consultative exercise leading to the co-creation of animal data governance that truly captures ethical issues. This future research can include how these findings individually can or do shape animal data governance.

It is furthermore worth underlining that the existence of ethically sensitive data governance will not make the underlying ethical issues go away. Many of these issues touch on deep convictions of who we are and what we as humans can or should do. Such convictions do not change quickly and different views will remain. But, as indicated earlier, ethical pluralism does not need to be seen as a problem but can be celebrated as an expression of human diversity. What counts is that we find productive ways of dealing with issues. Ethically informed animal data governance can be one mechanism that allows us as researchers, as citizens of different countries, holders of different convictions to come together and have productive conversations about how to understand and deal with our different worldviews. If it achieves this, then this will not only strengthen neuroscience with all its concomitant benefits but also show how science can play an important role in tackling the broader ethical and social questions that our increasingly globalized world faces.

## Data availability statement

The original contributions presented in this study are included in the article/supplementary material, further inquiries can be directed to the corresponding author.

## Ethics statement

Ethics approval was obtained for this research from De Montfort University Ethics Review Committee. Information sheet and an informed consent form were then emailed to participants.

## Author contributions

DE: conceptualization, writing, data analysis, original draft/review, and editing. GO: conceptualization, writing, and data collection. WK: conceptualization and writing. BS: conceptualization, writing, and funding. All authors contributed to the article and approved the submitted version.

## Footnotes

1
https://gdpr-info.eu/

2
https://www.hhs.gov/hipaa/index.html

3
https://www.priv.gc.ca/en/privacy-topics/privacy-laws-in-canada/the-personal-information-protection-and-electronic-documents-act-pipeda/
